# Collagen Osteoid-Like Model Allows Kinetic Gene Expression Studies of Non-Collagenous Proteins in Relation with Mineral Development to Understand Bone Biomineralization

**DOI:** 10.1371/journal.pone.0057344

**Published:** 2013-02-27

**Authors:** Jérémie Silvent, Nadine Nassif, Christophe Helary, Thierry Azaïs, Jean-Yves Sire, Marie Madeleine Giraud Guille

**Affiliations:** 1 UMR 7574, Chimie de la Matière Condensée de Paris, Ecole Pratique des Hautes Etudes, Université Pierre et Marie Curie, Paris, France; 2 UMR 7138, Equipe Evolution et développement du squelette, Université Pierre et Marie Curie, Paris, France; National University of Ireland, Galway (NUI Galway), Ireland

## Abstract

Among persisting questions on bone calcification, a major one is the link between protein expression and mineral deposition. A cell culture system is here proposed opening new integrative studies on biomineralization, improving our knowledge on the role played by non-collagenous proteins in bone. This experimental *in vitro* model consisted in human primary osteoblasts cultured for 60 days at the surface of a 3D collagen scaffold mimicking an osteoid matrix. Various techniques were used to analyze the results at the cellular and molecular level (adhesion and viability tests, histology and electron microscopy, RT- and qPCR) and to characterize the mineral phase (histological staining, EDX, ATG, SAED and RMN). On long term cultures human bone cells seeded on the osteoid-like matrix displayed a clear osteoblast phenotype as revealed by the osteoblast-like morphology, expression of specific protein such as alkaline phosphatase and expression of eight genes classically considered as osteoblast markers, including *BGLAP, COL1A1*, and *BMP2*. Von Kossa and alizarine red allowed us to identify divalent calcium ions at the surface of the matrix, EDX revealed the correct Ca/P ratio, and SAED showed the apatite crystal diffraction pattern. In addition RMN led to the conclusion that contaminant phases were absent and that the hydration state of the mineral was similar to fresh bone. A temporal correlation was established between quantified gene expression of DMP1 and IBSP, and the presence of hydroxyapatite, confirming the contribution of these proteins to the mineralization process. In parallel a difference was observed in the expression pattern of *SPP1* and *BGLAP,* which questioned their attributed role in the literature. The present model opens new experimental possibilities to study spatio-temporal relations between bone cells, dense collagen scaffolds, NCPs and hydroxyapatite mineral deposition. It also emphasizes the importance of high collagen density environment in bone cell physiology.

## Introduction

Bone formation is a multistep process starting by gene expression of osteoblasts leading to protein synthesis and formation of a hydrated gel, called osteoid. At first non mineralized, the osteoid tissue is essentially composed of collagen fibrils together with low fractions of non-collagenous proteins (NCPs). The three dimensional lattice of the osteoid, together with NCP activity, then contribute to specific apatite mineral deposition [1]. *In vivo* the expression patterns of several acid-rich secretory calcium-binding phosphoprotein (SCPP) genes, coincide with the bone mineralization process [2], [3]. Moreover, correlations have been established between the dysfunction of some acid-rich SCPPs and impaired apatite deposition [4], [5]. However, *in vivo*, the exact role played by each actor, collagen scaffold, NCPs and circulating fluids, remains controversial, leading scientists to set up more controlled *in vitro* biomineralization experiments.

At first, biologists analyzed osteoblast activities of cells seeded on glass or plastic, easy and convenient two-dimensional cell culture models [6]–[10]. These experiments allowed to study the osteoblast phenotype through the production of alkaline phosphatase, bone Gla protein (osteocalcin), various acid-rich SCPPs, and mineral deposition. However they remained far from *in vivo* conditions. In addition, cell culture scaffolds used in cell biology are sponges [11], hydrogels [12], [13], cements [14], or demineralized bone matrix [15], [16].

In parallel, chemists and material scientists introduced mineralization studies on acellular models, either in absence [17], [18] or presence of collagen [19], [20]. In these experiments, the concentration of ionic precursors, used to obtain a bone-like apatitic phase, always appeared higher than in circulating fluids when measured *in vivo*. Altogether these experiments remain far from physiological conditions. Indeed, bone mineralization is the result of complex interactions between bone cells and the surrounding extracellular matrix. The latter consists of a dense collagen network impregnated with ionic precursors and numerous NCPs, including specific mineralization proteins.

The know-how in producing fibrillar collagen scaffolds in the concentration range and fibril diameter found *in vivo* initiated cell/matrix interaction studies [21]. Transformed osteoblast cell lines seeded at the surface of dense 40 mg/mL collagen matrices were shown to proliferate and express alkaline phosphatase, a marker of differentiated osteoblasts [22]. However, use of immortalized cells, remained unsatisfactory as cell/matrix adhesion and/or protein gene expression can be, in these conditions, different from the behavior of cells *in vivo* [23].

In the present study human primary pre-osteoblasts were seeded on dense collagen matrices, displaying parameters chosen as osteoid-like regarding collagen fibril diameter, collagen matrix density and culture medium conditions. In a long-term culture of 60 days, pre-osteoblasts proliferated, then differentiated into active osteoblasts, in which gene expression of mineralization proteins were quantified and bone-like hydroxyapatite production demonstrated. The present work establishes a clear kinetic correlation between gene expression levels of mineralization proteins and apatite formation steps, validating the osteoid-like cell culture system. This model offers a promising and adaptable tool to modulate and experiment the different parameters implied in bone mineralization.

## Materials and Methods

### Collagen 3D Matrices

A solution of type I collagen at 3 mg/mL in 0.1% acetic acid was prepared as previously described [22], [24]. Briefly, collagen was extracted from rat tail tendon. After a washing step with phosphate-buffered saline (PBS), tendons were solubilized in 0.5 M acetic acid and the solution was clarified by centrifugation (21,000 rpm, 2h, 11°C). The supernatant was selectively precipitated with 0.3 M and 0.6 M of NaCl in order to remove proteins other than type I collagen, then collagen, respectively, by two centrifugations (21,000 rpm, 3h, 11°C then 4,400 rpm, 45 min, 11°C). The pellets were solubilized in 0.5 M acetic acid and dialyzed against 0.1 M acetic acid in order to desalt solution. A final centrifugation was done (21,000 rpm, 4h, 11°C) and the concentration adjusted to a final stock concentration of 3 mg/ml. The final concentration of type I collagen solution was estimated by hydroxyproline titration [25]. To obtain a solution of collagen at 40 mg/mL, the concentration process was carried out by controlled evaporation in sterile condition [26]. Acid soluble collagen at 3 and 40 mg/mL were placed in a sealed glass chamber in presence of ammonia vapors to induce fibrillogenesis and obtain three-dimensional fibrillar collagen matrices.

### Cell Culture

Primary human pre-osteoblasts were obtained from the knees (cancellous bone) of three healthy donors (male patients, aged 50–60 years) (Promocell) at passage 2. The manufacturer certified the absence of pathology on the tissue obtained from knee surgery. The cells were grown in Dubelco's Modified Eagle Culture Medium (DMEM, Gibco) containing 10% Fetal Calf Serum (Gibco), 100 U/ml penicillin (Gibco), 100 µg/ml streptomycin (Gibco), 0.25 µg/ml Fungizone (Gibco) and 10^−8^ M dexamethasone [27]. 75 cm^2^ culture flasks were kept at 37°C in a humidified atmosphere of 95% air/5% CO_2_ At confluence, adherent osteoblasts were enzymatically removed by treatment with 0.1% trypsin and 0.02% EDTA (both Gibco). Osteoblasts were used at passage 7 for the experiments in order to have a sufficient number of cells. The osteoblasts were seeded at the surface of the matrices at the density of 35,000 cells/cm^2^, and were grown in the same cellular medium, which was supplemented with 50 µg/ml ascorbic acid (VWR) and 3 mM NaH2PO4 (Sigma). Cells were cultured on matrices for 28 days and kept at 37° in a humidified atmosphere of 95% air/5% CO2. The matrices seeded with osteoblasts were sampled at days 7, 14, 21 and 28 for each study. Each experiment was sampled in triplicate.

### Cell Adhesion and Viability

The matrices were collected after 30 minutes, 3 hours, 6 hours, 12 hours and 72 hours, washed three times with PBS and fixed with 4% paraformaldehyde in PBS. After another washing in PBS, the cells were permeabilized in a Tween solution (0.05% Tween-20 PBS, 1% bovine serum albumin in PBS) for 20 min and stained for 10 min with DAPI diluted 1/50,000 in PBS. Remaining adherent cells were counted in a fluorescence microscope (AXIO 100 Zeiss), over a total of seven random fields (×10 magnification, 867×650 µm). For each time point, samples were analyzed in triplicate and the results were given as the mean of the remaining cells on three samples.

A MTT assay (reduction of 3-(4,5-dimethylthiazol-2-yl)-2,5-diphenyltetrazolium bromide to a dark-blue Formazan product) was used to assess the metabolic activity of osteoblasts cultured on collagen matrices [28]. This is related to the cell viability as viable cells metabolize MTT by the activity of mitochondria dehydrogenase. In this experiment, cells were cultured in serum-free condition. At days 7, 14, 21 and 28, the matrices were washed, then incubated with a 5 mg/ml MTT solution for 4 h. After three washes with PBS, Formazan was dissolved with dimethylsulfoxide (DMSO) and the absorbance of solution was measured at 570 nm with a Ultrospec 1100 Pro spectrophotometer (Amersham Biosciences). In order to normalize the results on cell number, a standard curve was established using several cell densities (from 15,000 to 150,000). Cell density was determined for each sample and expressed as a percentage of the initial number of pre-osteoblasts seeded on the matrices.

### Histology: Hemalun, von Kossa and Alizarin Red Staining

Samples were fixed in 4% paraformaldehyde and embedded in paraffin for sectioning. Ten µm-thick serial sections obtained perpendicularly to the cell layer, were dewaxed, rehydrated and stained specifically by Hemalun, staining the cell nuclei, von Kossa, identifying divalent ions and Alizarin Red S, specific of calcium ions. The slides were rinsed, dehydrated and mounted for observation with an optical microscope (Nikon E600 POL) or an epifluorescence microscope (AXIO 100 Zeiss).

### Alkaline Phosphatase Immunocytochemistry

The paraffin sections were dewaxed, rehydrated, washed for 10 min in PBS, and incubated for 6 min with 0.2% pepsin in 10% acetic acid (v/v). After rinsing in PBS, the sections were incubated for 30 min with 1% glycin PBS solution (v/v). After another washing in PBS, the sections were incubated for 60 min, at room temperature, with a blocking solution (0.05% Tween-20 PBS, 1% bovine serum albumin -BSA- and 10% FBS in PBS). The slides were covered with primary antibody against human alkaline phosphatase (A-2951 Sigma) diluted 1/100 (v/v) with the diluting solution (0.05% Tween-20 PBS, 1% BSA) and incubated overnight, at room temperature, in a moist chamber. Then, the slides were rinsed three times in PBS for 10 min, covered with the secondary antibody [anti mouse, cross-linked with rhodamine (Molecular Prob), diluted 1/400 (v/v)], and incubated for 90 min, at room temperature, in a moist chamber. After three rinsing in PBS for 10 min, the slides were incubated for 10 min with DAPI diluted 1/50,000 in PBS. Finally, the sections were washed three times with PBS, mounted, observed in a fluorescence microscope (AXIO 100 Zeiss) and photographed with a CCD camera (AxioCam MRm Zeiss).

### Scanning Electron Microscopy and Energy-dispersive X-ray Spectroscopy

At each time point, the samples were fixed in 3.6% glutaraldehyde in a cacodylate/saccharose buffer solution (0.05 M/0.6 M–pH 7.4). After critical point drying, the samples were sputter-coated with a gold layer of 10 nm and observed in a Hitachi S-3400 N operating at 12 kV.

An energy-dispersive X-ray (EDX) analysis was used to examine regions, in which deposits were identified, on samples at day 60. This analysis was carried out at day 60. The EDX instrument X-Max (Oxford Instruments) was attached to the scanning electron microscope Hitachi S-3400 N operating at 12 kV, and the Oxford Microanalysis Group XAN.70 software was utilized for this analysis.

### Transmission Electron Microscopy and Selected Area Electron Diffraction

Samples at day 14 were fixed in 2% osmium tetroxyde in a cacodylate/saccharose buffer solution (0.4 M/0.6 M-pH 7.4), rinsed, dehydrated and embedded in araldite. Ultrathin sections (100–200 nm) were contrasted by uranyl-acetate. Osmium and uranyl acetate were not added at day 60, to avoid artifacts. Sections were observed in a Tecnai spirit G2 at 120 kV.

### Reverse Transcriptase and Quantitative Real-time RT-PCR

Total RNA were isolated from matrices using RNeasy Fibrous mini kit (Qiagen). For each sample, RNAs were reversed transcribed into complementary DNA (cDNA) by a RevertAid™ H Minus M-MulV RT enzyme (Invitrogen) at 37°C. Forward and reverse primers for *GAPDH*, *BGLAP*, *IBSP*, *SPP1*, *MEPE, DMP1, ALPL, SPARC,PPARγ2, MYOD, BMP2, ANK* and *COL1A1* were designed using Primer 3 [29] (Table S1). The target genes were amplified in a thermal cycler (Mastercycler pro, Eppendorf). Cycling conditions were initial denaturation at 94°C for 2 min followed by 35 cycles, each cycle consisting of 30 sec of denaturation at 94°C, 45 sec of annealing at 60°C and, 45 sec of elongation at 72°C. Final elongation was for 20 min at 72°C. PCR products were observed in an analyser Gel Doc (BIORAD) after migration in a 1.5% agarose gel with ethidium bromide.

Gene expression was quantified using real-time reverse transcriptase PCR in a Light Cycler 480 detection system (Roche). The Light Cycler FastStart DNA Master plus SYBR Green I kit (Roche) was used for cDNA amplification, with a similar DNA concentration for all the qPCR. Cycling conditions were initial denaturation at 94°C for 5 min, followed by 45 cycles, each cycle consisting of 10 sec of denaturation at 94°C, 15 sec of annealing at 60°C and 15 sec of elongation at 72°C. Then, for each gene a melting curve was obtained by increasing the temperature from 65°C to 97°C, at a rate of 0.11°C/s. The efficiency (E) of the target primer pairs was measured by producing a curve based on serial dilution of cDNA. Relative expression was calculated using a mathematical model [30] and using the housekeeping gene GAPDH as normaliser. Indeed, this gene is constitutively expressed with the same level in all cells. Hence, we indirectly took into account the RNA amount, which is directly related to the number of living cells. Therefore, the gene expression values do represent all living cells at each time point. For each target gene, a ratio was calculated by comparison with a calibration point, which was the first expressed time point. The value 1 was arbitrary given to this calibration point. For each time point, our quantification was made in triplicate and the results presented as the mean relative expression ± standard deviation.

### Nuclear Magnetic Resonance

Solid state nuclear magnetic resonance (NMR) experiments were realized on sample at day 60. Magic angle spinning (MAS) spectra were acquired at a frequency of 8 kHz, with samples packed into 4 mm zirconia rotors. ^1^H-^31^P cross polarization (CP) experiments were performed on a Avance 300 Bruker spectrometer operating at frequencies of 300.13 MHz (^1^H) and 121.50 MHz (^31^P). The contact time (CT) and the recycle delay (RD) were set at 10 ms and 1 s, respectively. Two dimensional ^1^H-^31^P heteronuclear correlation (HETCOR) was performed with the following parameters RD = 1 s, CT = 10 ms, 1760 transients for each 128 t_1_ increment. ^1^H and ^31^P chemical shifts were referenced (δ = 0 ppm) to TMS and to 85%w aqueous H_3_PO_4_, respectively.

### Statistical Analysis

Statistical significance was determined using the Shapiro-Wilk test, followed by a Student parametric test or a Mann-Whitney-Wilcoxon non-parametric test if the differences were significant or not, respectively. Differences were considered significant when the p-value was <0.05. Data are given as mean ± standard deviation.

## Results and Discussion

**Figure 1:**
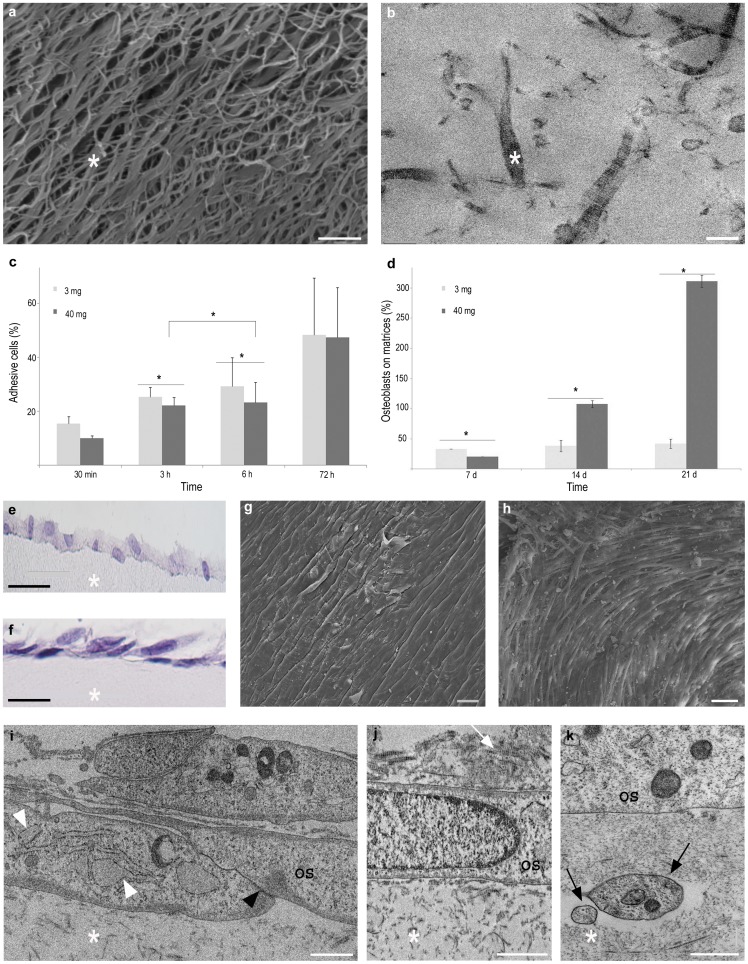
Matrix description and behavior of human primary osteoblasts seeded on an osteoid-like matrix. SEM (a) and TEM (b) views of the dense, 40 mg/mL, fibrillar collagen matrix (*). Scale bars: a: 1 µm, b: 100 nm. c, d: Cell adhesion over 72 hours and cell viability over 21 days analyzed by cell counting and MTT test, respectively. * = p<0.05. e–k: Osteoblast morphology. e, f: Hemalun staining of 5 µm-thick sections at 14 and 28 days, respectively; g, h: SEM observations at 14 and 28 days; i–k: TEM observations at 14 (i, j) and 60 days (k). i: The osteoblasts (OS) are organized into several layers and show a well-developed network of rough endoplasmic reticulum (white arrowheads) and are linked by means of tight junctions (black arrowhead); j: neosynthesized collagen (white arrow); k: cell extensions penetrating superficial region of the dense matrix (black arrows). Scale bars: e: 50 µm, f: 20 µm, g: 30 µm, h: 25 µm, i and j: 1 µm, k: 0.5 µm.

The behavior of human primary osteoblasts seeded on osteoid-like matrices, *i.e.* dense collagen scaffolds at 40 mg/mL, was compared to that of the same cells seeded on loose collagen matrices at 3 mg/mL, classically used in previous experiments [12], [13]. Macroscopically, the 40 mg/mL collagen matrices are opaque and rigid. Scanning electron microscopy (SEM) reveals a macroporous network, in which the fibril diameter ranges from 50 to 80 nm (Fig. 1a). This organization is confirmed by transmission electron microscopy (TEM), which reveals the typical collagen striated fibrils (Fig. 1b). On both matrices, osteoblasts adhesion was not statistically different at 30 minutes and 72 hours, at which a maximum of cell adhesion was obtained. In contrast, at 3 and 6 hours, the adhesion on loose matrices was higher than on dense matrices (Fig. 1c; Table S2). From 7 to 21 days on the dense matrices, osteoblast viability increased quasi exponentially and their number reached more than 300% the number of cells that adhered at 72 hours (Fig. 1d). In contrast, on the loose matrices, during the same length of time the number of osteoblasts increased by 40% only. These findings show that at the highest collagen matrix concentrations the cell viability increases. These results can be explained by an enhancing effect on osteoblasts of the dense collagen organization, in relation with the improved mechanical properties of the 40 mg/mL osteoid-like matrices [31]. The following data will thus concentrate on osteoblasts seeded on 40 mg/mL osteoid-like matrices. The osteoblasts morphology was compared at different culture time points in order to characterize the primary cell culture model on dense collagen matrices. Hemalun, a nuclear histological stain, revealed the cuboidal shape of the osteoblasts, from day 1 to day 14 (Fig. 1e). Such morphology is similar to that of highly active osteoblasts in vivo [32]. Indeed, bone ultrastructure studies all show that the cells are aligned on the border of the matrix, in an epithelial-like manner. From day 21 to day 60, the cells appeared flattened (Fig. 1f), a morphology similar to bone lining cells in vivo [33]. At day 14, SEM observations showed that the osteoblasts have covered the matrix surface (Fig. 1g). The cell layer had a similar aspect at day 28, although the osteoblasts increased in number (Fig. 1h). Between days 14 and 28, the cells have acquired an elongated shape and their largest width reduced from 20 µm (±5 µm) to 5 µm (±1 µm), respectively. From a histological point of view, at day 28 two to three osteoblast layers cover the matrix (Fig. 1f), an organization that was confirmed using SEM (Figure S1). Regardless the culture time, cells were aligned on the matrix and did not penetrate into the collagen network which is representative of in vivo tissue architecture. Indeed in bone, only some osteoblasts become entrapped into the collagen matrix and this induces their differentiation into osteocytes, but at this time the surrounding collagen concentration is much higher.

Moreover, TEM observations revealed the presence of tight junctions between adjacent osteoblasts, as well as a well-developed network of rough endoplasmic reticulum and numerous vesicles (Fig. 1i). These features are indicative respectively of cell-cell adhesion and active protein synthesis [34]. From day 14 onwards, patches of newly synthesized collagen fibrils appear, located in the vicinity of the osteoblasts, which are distinguishable from the fibrils in the subjacent matrix by their very uniform diameters and banded features (Fig. 1j). At day 60 cytoplasmic extensions from the osteoblasts have penetrated the dense collagen matrix (Fig. 1k). Hence the behavior and morphology of human primary osteoblasts on dense collagen matrices are reminiscent of osteoblasts *in vivo*. The present work exploits, through a two-month study, cells seeded at a pre-osteoblastic stage, that differentiate into distinctive early osteoblasts characterized by a cuboidal shape and finally become elongated cells, characteristic of mature osteoblasts *in vitro* [35]. The osteoid-like culture model thus offers long-term kinetic studies of osteoblasts functioning at all integration levels.

**Figure 2:**
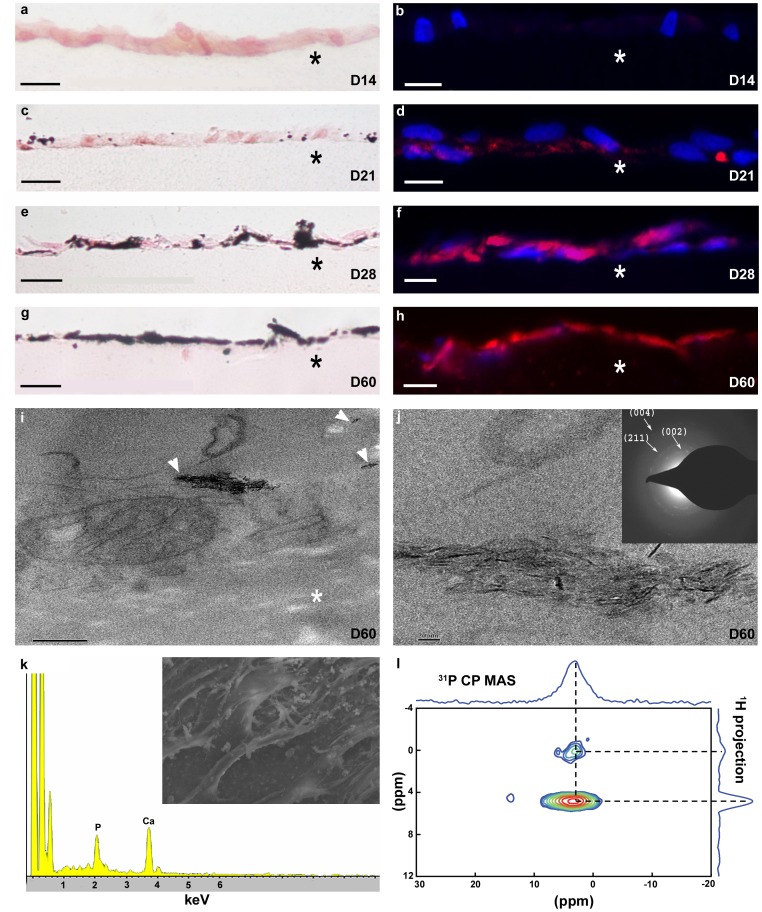
Demonstration of bone-like apatite formation at the surface of the osteoid-like matrix. a–h: Paraffin sections of matrices from 14 to 60 days. **a, c, e, g:** Staining with von Kossa indicates the presence of phosphate. **b, d, f, h:** Staining with alizarin red observed with epifluorescence (calcium in red, nucleus in blue) demonstrates the presence of calcium. Scale bars: **a, e, f:** 20 µm, **b, d**: 25 µm, **c:** 15 µm, **g**, **h:** 30 µm. **i:** Mineral deposits (arrowheads) observed in the osteoblast vicinity at day 60. **j:** High magnification showing apatite platelets. Scale bars: **i:** 200 nm, **j**: 20 nm. **Inset**: Electron diffraction pattern corresponding to the selected area. **k:** Calcium phosphate ratio analyzed by EDX coupled with SEM (**inset**) at day 60. **l**: 1D ^31^P CP MAS and 2D ^1^H-^31^P HETCOR experiment recorded at day 60, showing evidence of the biomimetic hydroxyapatite NMR fingerprint [correlation resonances between the phosphate groups δ(^31^P) = 3 ppm and the hydroxyls and water δ(^1^H) = 0 ppm and 4.8 ppm, respectively].

Because one of the major functions of mature osteoblasts consists in regulating the mineralization of the collagen matrix, the presence of a mineral phase was performed using a broad-spectrum of methodologies. Investigations from a histological point of view were realized at days 14, 21, 28 and 60. Paraffin sections were stained with von Kossa (Fig. 2a, c, e, g), that reacts with phosphate, and with alizarin red (Fig. 2b, d, f, h) that reveals the presence of calcium ions. Both methods showed the absence of a mineral phase at day 14 (Fig. 2a, b). At day 21, a weak labeling was observed at the level of the osteoblast layer (Fig. 2c, d), and the signal increased from day 28 (Fig. 2e, f) to day 60 (Fig. 2g, h). The staining was always restricted to the osteoblasts level above the dense matrix surface. However, it is worth noting that von Kossa and alizarin red staining can give inconclusive results [36], [37] when phosphate or calcium are uptaken by the matrix but are independent of mineral deposition [38]. Therefore, other methods further analyzed the mineral deposits observed in our model. At high TEM magnification (Fig. 2i, j), either dispersed or agglomerated electron-dense platelets were found in the neo-synthesized collagen close to the osteoblasts, confirming histological observations. This suggests that mineral nucleation occurred in the recently deposited extracellular matrix. TEM observations show platelets in the size range of 24±6 nm in length and 2.6±0.6 nm in thickness, values similar to bone apatite crystals [1]. However the amount of mineral platelets observed in TEM seemed low compared to histology data. The presence of holes in the sections probably corresponded to crystals, removed during sectioning in relation with a poor embedding of the mineral phase.

Thus, to evaluate the mineral phase further, we measured the amount of mineral phase in the matrices by quantitative TGA analyses at day 28 and 60 (Figure S2). By weighing the residual mass observed at 800°C, we obtained the percentage of the hydroxyapatite phase, hence at D28, the mass obtained was ±2%, and at D60, ±21%. These data confirm an increase in the mineral content throughout the 60 days culture, showing cells have differentiated into mature osteoblasts. The value at D60 appears similar to demineralized bone when remineralized by a treatment with poly-aspartic acids [39]. The mineral content did remain lower to that found in native bone but correlates with a mineral distribution observed at the surface of the matrices.

The corresponding selected-area electron diffraction (SAED) pattern (inset Fig. 2j) is typical of hydroxyapatite with distinct (002), (211), and (004), diffraction spots. The orientation of the (002) diffraction spots indicates a coalignment of the c-axis of the crystallites with the long axis of the collagen fibrils. Calcium and phosphorus peaks are detected by energy-dispersive X-ray (EDX) in SEM (inset Fig. 2k) at the surface of the matrices (n = 3) with an average Ca/P ratio of 1.51. In the absence of calcium phosphate contaminant phases, this ratio may indicate the existence of either amorphous calcium phosphate in the presence of HA crystals or calcium-deficient HA (CDHA) [40]. Thus, in order to characterize the calcium phosphate mineral phase, ^31^P solid-state nuclear magnetic resonance (NMR) experiments were performed taking advantage of a method with no sample preparation artifacts (Fig. 2l). 1D ^31^P cross polarization (CP) magic angle spinning (MAS) spectrum displays a single resonance centered at 3.2 ppm similar to phosphate groups in bone apatite in terms of chemical shift and line width (4.1 ppm) [41]. The 2D ^1^H-^31^P HETCOR spectrum reveals that the phosphate resonance at δ(^31^P) = 3.2 ppm correlates both with a proton resonance at δ(^1^H) = 0 ppm characteristic of hydroxyls in the hydroxyapatite phase and a strong resonance at δ(^1^H) = 4.8 ppm corresponding to water molecules adsorbed onto the apatite crystals. These features are similar to those found in fresh sheep bone and in bovine cortical bone [42] reflecting the high hydration degree of the apatite platelets. It is worth noting that the line width associated to the ^31^P resonance of the hydrated domain (5.8 ppm) is significantly broader than the line width of the ^31^P resonance corresponding to the apatite core (3.3 ppm) as observed in fresh sheep bone. The discrepancy of the line widths can be explained by a wider distribution in ^31^P chemical shifts due to various chemical environments in the hydrated domain, a feature that was described as a disordered layer around either synthetic [43] and biological [44] carbonated apatite. Moreover, neither calcium phosphate phase described as precursor (such as OCP) nor contaminant (such as brushite) were detected. In conclusion, the mineral phase formed in the long term, in the upper layers of the osteoid-like matrix, shows the hallmark of native bone hydroxyapatite.

**Figure 3:**
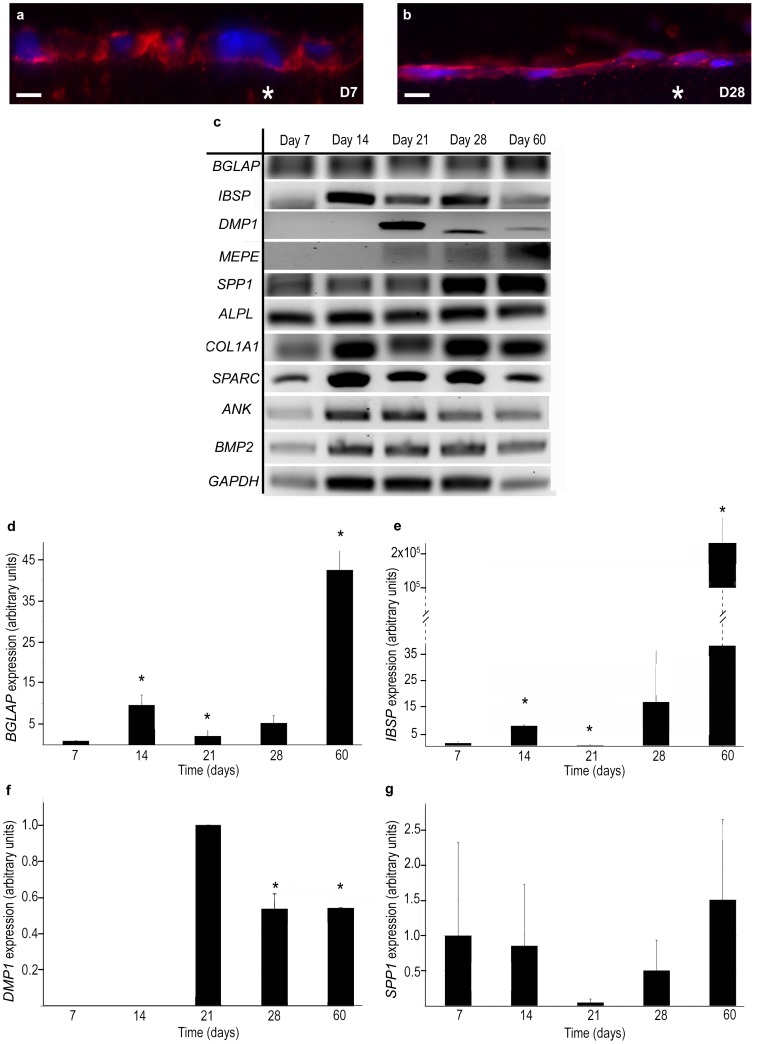
Protein and kinetic expression of various genes. a, b : Tissue-nonspecific alkaline phosphatase immunohistochemistry (nuclei in blue and alkaline phosphatase in red). At day 7 and day 28 alkaline phosphatase expression is localized over the cell membranes. Scale bars: **a:** 10 µm, **b:** 20 µm. **c:** Detection of *BGLAP*, *IBSP*, *DMP1*, *MEPE*, *SPP1*, *ALPL*, *COL1A1*, *SPARC*, *ANK*, *BMP2* and *GAPDH* transcripts using RT-PCR. **d–g:** Real time RT-PCR of four mineralization genes: *BGLAP* (**d**), *IBSP* (**e**), *DMP1* (**f)** and *SPP1* (**g**). * = p<0.05.

In order to demonstrate the osteoblast activity accurately and correlate it with mineral formation, we followed-up alkaline phosphatase (ALP = TNAP) expression together with the expression of significative protein genes, at all time points from preosteoblasts (day 7) to mature osteoblasts (day 60). ALP is considered as a marker of the osteoblast phenotype, its major function consisting in hydrolyzing inorganic pyrophosphate to ensure normal bone mineralization [45]. Using immunohistochemistry this protein was strongly detected at the osteoblast membrane from day 7 to 60 (Fig. 3a, b). In association with other proteins, ALP contributes to the deposit of hydroxyapatite platelets within the collagen matrix. The expression of ten genes coding for proteins either involved in the regulation of the mineralization process (*BMP2*, *BGLAP* and four acid-rich SCPPs, *DMP1, MEPE, IBSP* and *SPP1*) or characterizing osteoblast functioning (*COL1A1*, *ALPL*, *SPARC, ANK*) was studied using reverse transcriptase polymerase chain reaction (RT-PCR) (Fig. 3c). Eight of them, *BGLAP*, the gene coding for the bone-gla protein ( = OCN, osteocalcin), *IBSP,* the gene for the integrin-binding sialoprotein ( = BSP, bone sialoprotein), *SPP1*, the gene coding for the secreted phosphoprotein 1 ( = OPN, osteopontin), *COL1A1*, the gene coding for the collagen pro-alpha 1 chain, *ALPL*, the gene encoding the tissue-nonspecific alkaline phosphatase, *BMP2*, encoding bone morphogenetic protein 2, *ANK*, coding progressive ankylosis, and *SPARC*, encoding the secreted protein acidic and rich in cysteine ( = osteonectin), were found expressed over all time points of the experiment. *In vivo*, these eight genes are known as being expressed in early osteoblasts (*i.e*. cells producing the osteoid tissue) and in mature osteoblasts (*i.e*. cells contributing to bone matrix mineralization) [4]. The two other target genes, *DMP1*, the gene coding for dentin matrix acidic phosphoprotein 1, and *MEPE*, the gene for matrix extracellular phosphoglycoprotein, were only expressed from day 21 to day 60. DMP1 is known to be specifically expressed by mature osteoblasts [46], [47] and MEPE by mature osteoblasts entering the differentiation way towards the osteocytes and by differentiated osteocytes [48]. In order to test the purity of our culture system, we looked for the expression of *PPARγ2*, an adipocyte marker, and of *MYOD,* a myocyte marker. These genes were not found expressed in our culture. We further chose to quantify the expression of five mineralizing proteins genes using real time RT-PCR (normalized with the housekeeping gene *GAPDH*) in order to correlate their expression levels with the various steps of mineral phase (hydroxyapatite) formation. Although *MEPE* expression was detected in RT-PCR, gene expression was too low to be quantified. *BGLAP* and *IBSP* expressions increased significantly between day 7 and 14, decreased significantly from day 14 to 21, and increased significantly again from day 21 to 60, at which *BGLAP* expression was 20 times higher than at day 21. At the same time, the level of *IBSP* expression reached 7 million times the value at day 21 (Figs. 3d, e). *SPP1* expression did not show a significant difference during the experiment (Fig. 3g). As previously observed with RT-PCR experiment, *DMP1* expression was detected at day 21, then halved significantly at day 28 and remained unchanged until day 60 (Fig. 3f). Therefore, at day 21, when the first mineral phase is detected at the surface of the osteoid-like matrix, *DMP1* expression is high, a correlation which supports the role played by DMP1 in the early apatite nucleation steps by concentrating mineral ions at the protein binding sites [49], [50]. When mineral maturation starts at day 28, the expression of *IBSP* increases in an exponential way up to day 60, a correlation which comforts the role played by IBSP in apatite crystal growth [4], [51]. *SPP1* expression, which does not varies at all time points of our experiment, whether the mineral phase is present or not, does not seem to support the currently admitted role of SPP1 as preventing apatite formation [4], [52]. In the literature, *BGLAP* is generally known as inhibiting apatite nucleation [5], [53], but was also suggested as playing a role in the growth and morphology of apatite crystals [54]. In our dense collagen matrix model the high expression level of *BGLAP* at day 60 is in favor of a stimulating rather an inhibiting role of this protein. This contrasted result could be explained by the high collagen concentration in the matrices compared to previous experiments using loose matrices. A similar opposite effect of *IBSP* has been reported depending on a fluid or compact environment [55]. However, this hypothesis needs to be confirmed by functional experiments as, for instance, gene knockdown by small interfering RNA or overexpression of the protein. The observed correlations between gene expression levels and mineral nucleation and growth open here very new and promising research pathways to study experimentally many coordinated regulations of bone biomineralization.

### Conclusion

Taken together our results coincide to demonstrate the interest in using dense osteoid-like matrices, compared to other natural or synthetic scaffolds, in order to study osteoblast behavior and mineralization processes *in vitro*. Various collagen-based cell culture scaffolds were used in previous studies: they were sponges [11], hydrogels and demineralized bone matrices. Concerning sponge material, scaffolds have a low collagen concentration and are processed in absence of fibrils, making their structure completely different from that of bone tissues. Hydrogels are loose fibrillar collagen scaffolds possessing weak mechanical properties; they do not provide appropriate signals for cells to mimic physiological behavior [56]–[58]. The demineralized bone matrices used in the study of osteoblast maturation are not a controlled, reproducible system [15], [16]; neither the collagen concentration nor the structure of the matrix are exactly known, since they can vary with regard to skeleton region and can be modified during the demineralization process. In addition, in this model the demineralized bone matrix components are difficult to define; some proteins can remain adsorbed in the matrix after demineralization and then, be involved in osteoblast maturation.

Our experiments using primary osteoblasts validate the dense collagen scaffolds as appropriate osteoid models. Indeed their structure in terms of fibril diameter, concentration and 3D order are close to an early bone tissue. As a cell culture they provide data similar to those *in vivo* conditions, both in terms of apatite mineral deposition and expression of structural and mineralization protein genes. In the future, our model will allow to address different sorts of questions, whether considering *in vivo*-like cellular models or *in vitro* acellular conditions. As a function of different cell culture conditions, by modifying media components, the surrounding fluid composition or the culture pressure conditions this osteoid-like matrix will possibly establish new links between the expression of mineralization proteins by osteoblasts and various steps of apatite mineral formation, including the presence of crystalline or amorphous phases. In acellular conditions, this model will also open the possibility to test the specific role of proteins or peptides, by their injection in a dense collagen environment close to *in vivo* conditions, for example the action of acid-rich SCPPs involved in either in mineral nucleation [49], or inhibition [59]. This highly innovative and adaptable approach will allow to explore the role played by either by the cells, the matrix or the proteins during the successive events leading to bone mineralization.

## Supporting Information

Figure S1 **SEM observations of osteoblasts seeded on dense collagen matrices.**
(RTF)

Figure S2 **Thermogravimetric analyses of matrices at D28 and D60.**
(RTF)

Table S1 **Primer sequences used for PCR and real-time PCR analysis of the eleven genes, and amplicon lengths.**
(DOCX)

Table S2 **Osteoblast adhesion.**
(RTF)
