# Supplementary material for: Collagen Osteoid-Like Model Allows Kinetic Gene Expression Studies of Non-Collagenous Proteins in Relation with Mineral Development to Understand Bone Biomineralization
Source: PLoS One. 2013 Feb 27;8(2):e57344. doi: 10.1371/journal.pone.0057344 (PMC3583827; doi:10.1371/journal.pone.0057344)
Supplement: Figure S1 — SEM observations of osteoblasts seeded on dense collagen matrices. (RTF) [file pone.0057344.s001.rtf]

SEM observations of osteoblasts seeded on dense collagen matrices 

Fig. S1 : 
Osteoblast layers (arrowheads) at day 14 (a) and 60 (b). Collagen matrix (*). Scale bars: a : 2.5 µm, b : 5 µm.
