# Supplementary material for: Collagen Osteoid-Like Model Allows Kinetic Gene Expression Studies of Non-Collagenous Proteins in Relation with Mineral Development to Understand Bone Biomineralization
Source: PLoS One. 2013 Feb 27;8(2):e57344. doi: 10.1371/journal.pone.0057344 (PMC3583827; doi:10.1371/journal.pone.0057344)
Supplement: Figure S2 — Thermogravimetric analyses of matrices at D28 and D60. (RTF) [file pone.0057344.s002.rtf]

Thermogravimetric analyses of matrices at D28 and D60

Samples were analyzed on a thermo-microbalance instrument (NETZSCH STA 409PC). The measurement was performed from room temperature to 1000°C in an oxidizing atmosphere with a heating rate of 5°C/min.


Determination of the residual mass by TGA The thermogravimetrics curves of a. matrices at day 28 and b. matrices at day 60.
