# Supplementary material for: Collagen Osteoid-Like Model Allows Kinetic Gene Expression Studies of Non-Collagenous Proteins in Relation with Mineral Development to Understand Bone Biomineralization
Source: PLoS One. 2013 Feb 27;8(2):e57344. doi: 10.1371/journal.pone.0057344 (PMC3583827; doi:10.1371/journal.pone.0057344)
Supplement: Table S1 — Primer sequences used for PCR and real-time PCR analysis of the eleven genes, and amplicon lengths. (DOCX) [file pone.0057344.s003.docx]

**Primer sequences used for PCR and real-time PCR analysis of the eleven genes, and amplicon lengths.**

| **Gene name** | **GenBank Accession No** | **Primer Sequences**  **Above: forward; below: reverse** | **Amplicon length** |
| --- | --- | --- | --- |
| ***BGLAP*** | NM_199173.4 | GAGGGCAGCGAGGTAGTGAAG  CTCCTGAAAGCCGATGTGGT | **153** |
| ***IBSP*** | NM_004967.3 | TTCCAGTTCAGGGCAGTAGTGACTC  TCCATAGCCCAGTGTTGTAGCAG | **173** |
| ***SPP1*** | NM_001040058.1 | TCTAAGAAGTTTCGCAGACCT  CGGCTGTCCCAATCAGA | **149** |
| ***DMP1*** | NM_004407.3 | AGAAGCGAGCTTGATGACAACAAC  TGGACTCACTGCTGGGACCATCTAC | **187** |
| ***MEPE*** | NM_001184694.1 | GGGCCTGCCCATTCCTTCTCGT  ACCCCAGGAGCCTTTCCCTTGTG | **178** |
| ***GAPDH*** | NM_002046.3 | GAAGGTGAAGGTCGGAGTCAAC  CAGAGTTAAAAGCAGCCCTGGT | **71** |
| ***COL1A1*** | NM_000088.3 | GGCCAAGAGGAAGGCCAAGTCGAG  CGCCGGGGCAGTTCTTGGTCTC | **187** |
| ***ALPL*** | NM_000478.4 | TCAACACCAACGTGGCTAAGAATGTC  GGCTTTCTCGTCACTCTCATACTCCAC | **590** |
| ***SPARC*** | NM_003118.2 | ATGAGACAGAGGTGGTGGAAGAAACTG  CATCCCTCTCATACAGGGTGACCAG | **471** |
| ***ANK*** | NM_054027.4 | CATCGCTGCCGTCTTTCAC  CACATCCCACCAGGAAACTG | **206** |
| ***BMP2*** | NM_001200.2 | GTGGGAAAACAACCCGGAG  CAGTCCACCGCATCACAG | **279** |
| ***PPARγ2*** | NM_138712.3 | TGGTGACTTTATGGAGCCCAA  GGCAAACAGCTGTGAGGACTCAG | **220** |
| ***MYOD*** | NM_002478.4 | CTCTCTGCTCCTTTGCCAC  GAGTGCTCTTCGGGTTTCAG | **133** |
