# Supplementary material for: Collagen Osteoid-Like Model Allows Kinetic Gene Expression Studies of Non-Collagenous Proteins in Relation with Mineral Development to Understand Bone Biomineralization
Source: PLoS One. 2013 Feb 27;8(2):e57344. doi: 10.1371/journal.pone.0057344 (PMC3583827; doi:10.1371/journal.pone.0057344)
Supplement: Table S2 — Osteoblast adhesion. (RTF) [file pone.0057344.s004.rtf]

Osteoblast adhesion
	30 min	3 h	6 h	12 h	72 h	
3 mg/mL	10.03 ± 0.90	22.16 ± 2.96	23.19 ± 7.50	23.01 ± 7.36	47.31 ± 18.55	
40 mg/mL	15.43 ± 2.53	25.37 ± 3.42	29.31 ± 10.69	31.67 ± 11.20	48.21 ± 21.27	

At 30 min after seeding the adhesion tests showed that the number of cells attached on the loose matrices were about 15% (15.42 % ± 2.52%), and about10% (10.02 % ±0.9 %) on the dense matrices. At 3, 6 and 12 hours, a quite similar number of cells were observed on the dense matrices (22.15 % ± 2.96 %, 23.18 % ± 7.49 % and 23.01 % ± 7.495 %, respectively) and on the loose matrices (25.37 % ± 3.41 %, 29.31 % ± 10.59 % and 31.67 % ± 11.21 %, respectively). After 72 hours, the end of the experiment, a maximum of cell adhesion was obtained. About half of the cells adhered at the surface of both matrices (47.31% ± 18.51 % and 48.21 ±21.27, respectively). Osteoblast adhesion was not significantly different after 30 minutes and 72 hours. In contrast, at 3 and 6 hours, a significant difference was observed, with a higher adhesion on loose matrices than on dense matrices.
